# Turning radiology reports into epidemiological data to track seasonal pulmonary infections and the COVID-19 pandemic

**DOI:** 10.1007/s00330-023-10424-6

**Published:** 2023-11-20

**Authors:** Tobias Heye, Martin Segeroth, Fabian Franzeck, Jan Vosshenrich

**Affiliations:** 1 Department of Radiology, University Hospital Basel, Petersgraben 4, 4031 Basel, Switzerland; 2 Division of Infectious Diseases and Hospital Epidemiology, University Hospital Basel, Petersgraben 4, 4031 Basel, Switzerland

**Keywords:** Radiology, Report, Epidemiology, Influenza, Disease outbreaks

## Abstract

**Objectives:**

To automatically label chest radiographs and chest CTs regarding the detection of pulmonary infection in the report text, to calculate the number needed to image (NNI) and to investigate if these labels correlate with regional epidemiological infection data.

**Materials and methods:**

All chest imaging reports performed in the emergency room between 01/2012 and 06/2022 were included (64,046 radiographs; 27,705 CTs). Using a regular expression-based text search algorithm, reports were labeled positive/negative for pulmonary infection if described.

Data for regional weekly influenza-like illness (ILI) consultations (10/2013–3/2022), COVID-19 cases, and hospitalization (2/2020–6/2022) were matched with report labels based on calendar date. Positive rate for pulmonary infection detection, NNI, and the correlation with influenza/COVID-19 data were calculated.

**Results:**

Between 1/2012 and 2/2020, a 10.8–16.8% per year positive rate for detecting pulmonary infections on chest radiographs was found (NNI 6.0–9.3). A clear and significant seasonal change in mean monthly detection counts (102.3 winter; 61.5 summer; *p *< .001) correlated moderately with regional ILI consultations (weekly data *r *= 0.45; *p *< .001).

For 2020–2021, monthly pulmonary infection counts detected by chest CT increased to 64–234 (23.0–26.7% per year positive rate, NNI 3.7–4.3) compared with 14–94 (22.4–26.7% positive rate, NNI 3.7–4.4) for 2012–2019. Regional COVID-19 epidemic waves correlated moderately with the positive pulmonary infection CT curve for 2020–2022 (weekly new cases: *r *= 0.53; hospitalizations: *r *= 0.65; *p *< .001).

**Conclusion:**

Text mining of radiology reports allows to automatically extract diagnoses. It provides a metric to calculate the number needed to image and to track the trend of diagnoses in real time, i.e., seasonality and epidemic course of pulmonary infections.

**Clinical relevance:**

Digitally labeling radiology reports represent previously neglected data and may assist in automated disease tracking, in the assessment of physicians’ clinical reasoning for ordering radiology examinations and serve as actionable data for hospital workflow optimization.

**Key Points:**

• *Radiology reports, commonly not machine readable, can be automatically labeled with the contained diagnoses using a regular-expression based text search algorithm.*

• *Chest radiograph reports positive for pulmonary infection moderately correlated with regional influenza-like illness consultations (weekly data; r = 0.45; p < .001) and chest CT reports with the course of the regional COVID-19 pandemic (new cases: r = 0.53; hospitalizations: r = 0.65; p < 0.001).*

• *Rendering radiology reports into data labels provides a metric for automated disease tracking, the assessment of ordering physicians clinical reasoning and can serve as actionable data for workflow optimization.*

## Introduction

In most health care systems, information is encoded and transmitted through various forms of text-based digital reports. Albeit digital, reports allow for digital distribution but mostly do not contain machine-readable data elements [1]. Consequently, report content and implications for patient care need to be gauged manually. Digitally extracting report content, for instance labeling a chest radiograph “positive for pulmonary infection,” would provide a signal, often referred to as actionable data, that can be used for various purposes. These include notifications, patient management, resource allocation, and data analysis [2]. Although the International Classification of Diseases (ICD) is used to label diseases, coding is based on the entire patient pathway including multiple diagnostic tests and the physicians’ conclusion [3]. Additionally, it is not real time since coding is regularly performed for billing purposes later in the process.

In light of the sustained increase in imaging studies worldwide, it would be desirable to obtain an understanding how imaging studies are utilized for the detection of a suspected disease [4–7]. Therefore, it is necessary to calculate the “number needed to image,” a concept in radiology similar to the “number needed to treat” metric in patient care. It allows to assess how reasonable a test is ordered by referring physicians [8].

Another application of labeling radiology reports can be to track the course of the detected disease over time. A controversially discussed example is the Google search engine flu tracker, which was able to partially predict the seasonal flu in the USA based on search queries [9–11]. The respective health care costs of diseases like the seasonal flu and the newcomer COVID-19 however mandate to broaden the arsenal of disease tracking tools. Rendering radiology reports into a data source is a neglected field of research. Only a few approaches, using either manually extracted labels with limited sample sizes or natural language processing (NLP)–based labeling, have been published so far [7, 12–15]. To our knowledge, only one other group has investigated automated radiology report labeling on a large scale to detect pulmonary infections by means of natural language processing [16].

The main goal of our study was to label reports of chest radiographs and chest CTs for presence or absence of pulmonary infection according to its content, and to investigate potential applications for this kind of data source. We aimed to investigate if radiology report results match regional epidemiological data for pulmonary infections, to reveal the positive rates for chest radiographs and chest CT identifying pulmonary infections, and to calculate the number needed to image.

We hypothesized that radiology report labels could serve as epidemiological data to track the course of regional pulmonary infection occurrence, and would allow to calculate the number needed to image. This metric could be used to assess clinical reasoning of physicians when requesting imaging examinations in patients with clinically suspected pneumonia.

## Materials and methods

The local ethics committee of northwestern and central Switzerland (project ID 2022-01016) approved this study. Written informed consent was waived. There is no conflict of interest, and no financial support was received for this study.

### Study sample

All radiology reports (*n *= 91,751) for chest radiographs (CR, *n *= 64,046) and CT examinations including the chest (*n *= 27,705) performed in the emergency department University Hospital Basel, Switzerland, from 1/2012 to 6/2022 were retrieved from the radiology information system (RIS) and included in the study. There were no exclusions. The reports, written in German as reporting language, were stored in a database (Tableau version 2022.2.1) with the date of examination, type of examination, and modality as metadata. Since 2015, chest radiographs and chest CTs from our department are written using structured reporting templates. Both templates use subheadings addressing anatomical structures and regions, for instance “lung parenchyma” and “pleural space.” Chest radiograph templates combine the section findings and impression, whereas CT reports contain a separate findings section including the aforementioned subheadings and a dedicated impression section.

### Text processing

**Fig. 1:**
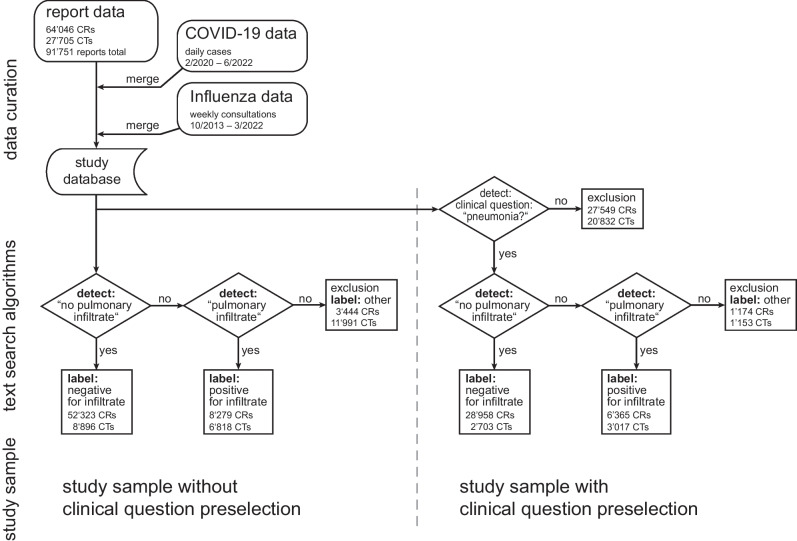
Flowchart of the study

The following steps were all performed using regular expressions, a rule-based syntax for processing of text data [2, 17]. Figure 1 illustrates data selection and text algorithm processing. Reports were split into the following segments based on keywords identifying section headings: clinical question, findings, and impression separate or combined. First, all reports were labeled positive or negative for pulmonary infections according to their findings. In a second approach, the clinical question segment was analyzed for grammatical variations of “pneumonia,” “infiltrate,” and “pulmonary infection” to identify examinations ordered by referring physicians for a suspected pulmonary infection (labeled “clinical question preselection”), resulting in 43,370 reports (36,497 chest radiographs and 6873 CTs). At our institution, infiltrate is exclusively used as an expression for a pulmonary infection.

In detail, the next text processing step for both approaches was to identify reports negative for a pulmonary infection by searching for variations of the expression “no pulmonary infiltrate/pneumonia present” (labeled “infiltrate negative”).

Reports not matching the preceding text search algorithm were processed to identify examinations positive for a pulmonary infection by detecting variations of the expression “pulmonary infiltrate/pneumonia present” (labeled “infiltrate positive”). The remainder of reports which did not match the preceding algorithms were labeled “other” and were excluded. These reports contain other findings and the text does not reference pulmonary infection. The algorithms’ output was tabulated next to the source text snippets, which allows for a rapid visual verification of the results and the algorithm’s accuracy. Furthermore, extensive sampling was performed on the labeling results to iteratively improve the algorithm and to include common spelling mistakes and grammatical text variations. Finally, a manual quality control, randomly sampling 5.4% of all reports (*n *= 5000), yielded an algorithm labeling accuracy of > 99.9%.

### Positive rate and number needed to image

The positive rate for pulmonary infections was calculated as the percentage of examinations positive for pulmonary infiltrates out of all examinations with a clinical question suspecting pneumonia. The number needed to image (NNI) is the number of examinations needed to achieve one positive examination.

### Influenza/COVID-19 data correlation

Weekly regional seasonal consultations for influenza-like illness from 10/2013 to 3/2022 assessed by the Sentinella general physician surveillance system were provided by the Swiss Federal Office of Public Health, Communicable Disease Division [18]. Regional COVID-19 epidemiological daily data (new cases and hospitalizations) from 2/2020 to 6/2022 were downloaded from the official Swiss health department website [19]. All data was merged into a single database matching based on calendar date. Chest radiograph data was correlated with influenza-like illness data for the time period 1/2012 to 2/2022 and chest CT data with COVID-19 data for the time period 3/2020 to 6/2022 because each type of examination was the institutional standard of care for the respective type of infection during the respective time periods.

### Data analysis

Trending line graphs over time, color coded by meteorological seasons spring (March–May), summer (June–August), autumn (September–November), winter (December–February), were created displaying positive and negative detection counts of pulmonary infections on chest radiographs and chest CTs. For each modality category (CR, CT) and time interval (day, week, month, season, year) absolute, mean and relative values expressed in percentages were calculated. The difference between groups of continuous variables was calculated using the Mann-Whitney *U* test. *P*-values < .05 were considered to represent a statistically significant difference. Linear best fit was derived using the ordinary least squares method. The correlation between the different data sources, report data, influenza-like illness, and COVID-19 cases was assessed using the Spearman’s correlation coefficient with 95% confidence intervals (CIs) calculated using the standard Fisher *z*-transformation [20]. Graphical and statistical analysis were performed using commercially available and open source software (Tableau 2022.2.1, Python Version 3.8.8 and scipy 1.10.1 [21]).

## Results

### Chest radiographs and pulmonary infections

Between 1/2012 and 2/2020, the monthly counts of pulmonary infection detection by chest radiographs ranged from 42 to 173 (clinical question preselection: 27 to 131) while chest radiographs negative for pulmonary infection ranged from 365 to 635 (clinical question preselection: 165 to 442). The per year overall positive rate for 2012 to 2020 ranged from 10.8 to 16.8% resulting in an NNI between 6.0 to 9.3 for pulmonary infections (clinical question preselection: yearly positive rate 14.6 to 22.6%; NNI: 4.4 to 6.8)

**Fig. 2:**
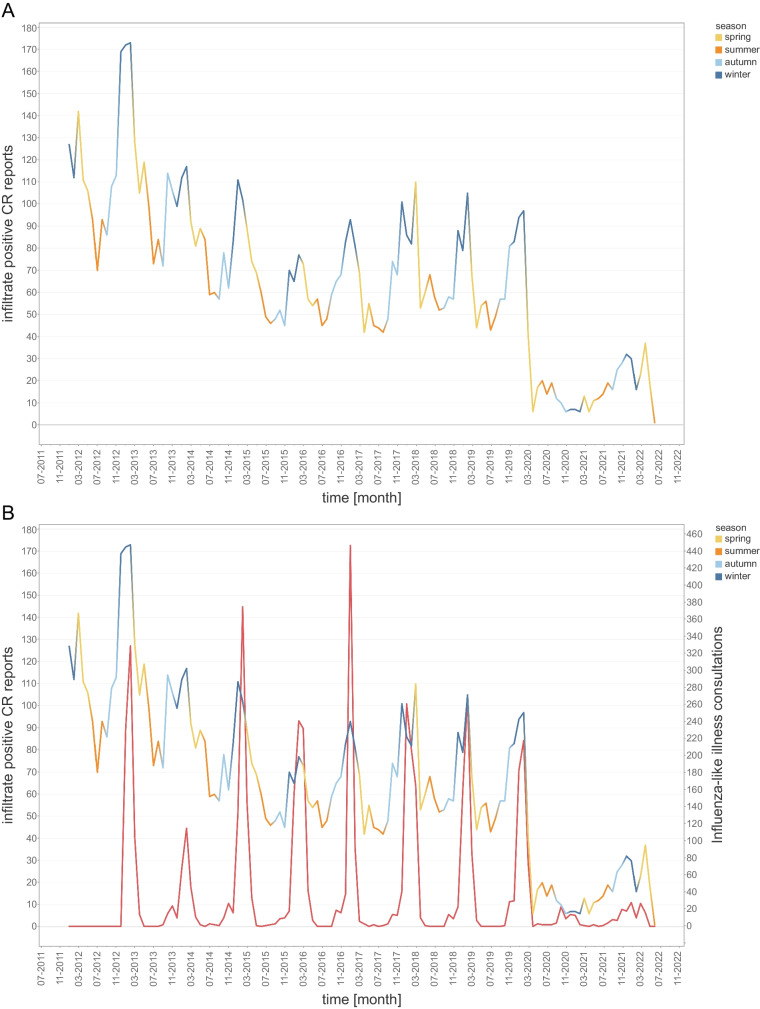
Infiltrate positive chest radiograph reports and Influenza-like illness consultations. **A** Chest radiograph reports positive for infiltrate detection from 1/2012 to 6/2022. **B** Sentinella data demonstrating number of consults for influenza-like illness superimposed with infiltrate positive chest radiograph data. Seasons, spring, summer, autumn, and winter are color coded. Please note the seasonality of re-occurring peaks of infiltrate positive chest radiographs in the winter months/season. The peaks match well with the Sentinella influenza data peaks

A recurring statistically significant seasonal difference was observed with a mean monthly detection count of 102.3 (range: 65 to 173) in the winter compared with 61.5 (range 42 to 99) in the summer months (overall mean for all seasons: 79.2; *p *< .001). Monthly positive rates were lower in the summer with 8.9 to 14.5%, NNI 6.9 to 11.2 (clinical question preselection: 11.6 to 20.2%; NNI: 5.0 to 8.6) compared with the winter months with 11.7 to 19.7%, NNI 5.1 to 8.5 (clinical question preselection: 16.0 to 27.2%; NNI: 3.7 to 6.3). This seasonality is visually apparent in Fig. 2A with periodic peaks in detected pulmonary infections occurring every year between December and February. The mean number of pulmonary infections detected by chest radiographs dropped to 28.6 per month in 2020, 15.8 in 2021, and 28.3 in 2022 (clinical question preselection: 20.8 in 2020; 9.4 in 2021; 19.3 in 2022). There was no visually evident seasonality for chest radiographs positive for pulmonary infections from 3/2020 to 6/2022.

### Chest radiograph correlation with influenza data

**Fig. 3:**
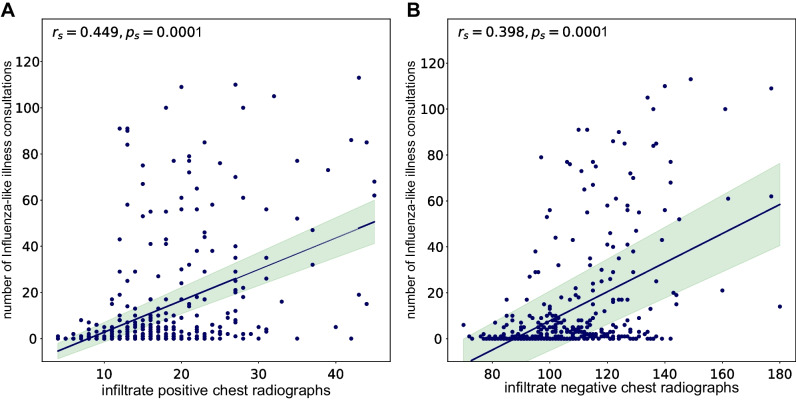
Influenza-like illness consultations—weekly data. Correlation between number of consults and infiltrate positive (**A**)/negative (**B**) chest radiographs per week demonstrated by a scatter plot with linear regression line

The observed seasonality of pulmonary infections detected by chest radiographs in the winter months correlates with the matched Influenza surveillance data, demonstrating matching peaks of influenza-like illness counts and a moderate correlation coefficient for weekly (*r *= 0.45; CI: 0.36–0.53; *p *< .001; Fig. 2B, Fig. 3) and monthly aggregation of data (*r *= 0.58; CI: 0.40–0.71; *p *< .001). The correlation with influenza data improved with preselection, when only examinations in which clinicians suspected a pulmonary infection were included (weekly *r *= 0.49; CI: 0.40–0.57; *p *< .001 and monthly *r *= 0.64; CI: 0.48–0.76; *p *< .001).

### Chest CTs and detection of pulmonary infections

A sharp increase in monthly counts of pulmonary infection detection by chest CT was seen starting in 3/2020 to 6/2022, ranging from 64 to 234 (clinical question preselection: 33 to 142). Chests CTs negative for pulmonary infection ranged between 121 and 236 (clinical question preselection: 38 to 127), yielding a yearly positive rate of 23.0 to 26.7% and a NNI of 3.7 to 4.3 (clinical question preselection: positive rate 38.4 to 44.5%; NNI: 2.2 to 2.6). In comparison, chest CTs positive for pulmonary infection detection between 2012 and 2019 ranged from 14 to 94 per month, resulting in a yearly mean positive rate of 22.4 to 26.7%, NNI 3.7 to 4.4 (clinical question preselection: per month count 1 to 21; positive rate 40.0 to 53.4%; NNI: 1.9 to 2.5).

### Chest CT correlation with COVID-19 data

**Fig. 4:**
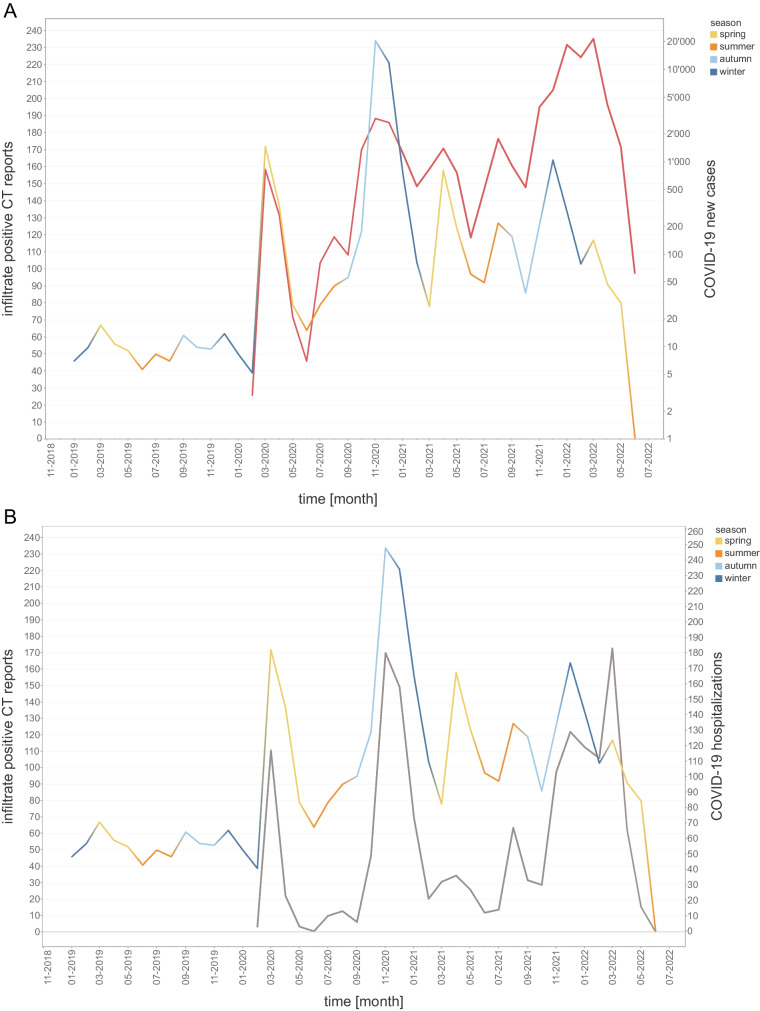
Infiltrate positive chest CT reports and COVID-19 new cases and hospitalizations. **A** Chest CT reports positive for infiltrate detection from 1/2019 to 6/2022 superimposed with regional COVID-19 new cases data (logarithmic scale). **B** Chest CT reports positive infiltrate detection from 1/2019 to 6/2022 superimposed with regional COVID-19 hospitalization data. Seasons, spring, summer, autumn, and winter are color coded. Please note matching peaks of infiltrate positive chest CTs with the various COVID-19 waves in new cases and hospitalizations. Please also note a relative divergence of decreasing pulmonary infections and increasing new COVID-19 cases when vaccines became available in early 2021

**Fig. 5:**
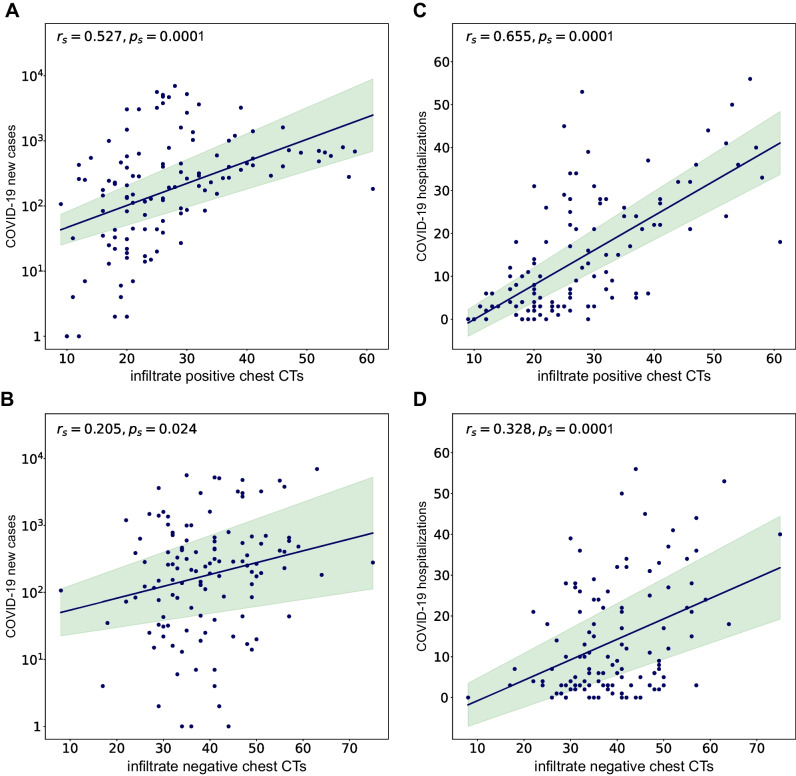
COVID-19 new cases and hospitalization—weekly data. Correlation between number of new COVID-19 cases (**A**, **B**) and hospitalizations (**C**, **D**) with infiltrate positive/negative chest CTs per week demonstrated by a scatter plot with linear regression line (*y*-axis logarithmic scale in **A**, **B**)

The trending line of chest CTs positive for pulmonary infections showed a distinct course and visually clearly followed the regional COVID-19 new cases and hospitalization curve (Fig. 4). The correlation between CTs positive for infiltrates and regional COVID-19 regional data was moderate for weekly aggregated data of new cases (*r *= 0.53; CI: 0.37–0.65; *p *< .001) and hospitalizations (*r *= 0.65; CI: 0.53–0.75; *p *< .001, Fig. 5). The daily correlation was fair (new cases *r *= 0.31; CI: 0.24–0.37; *p *< .001; hospitalizations *r *= 0.36; CI: 0.29–0.42; *p *< .001), but increased for monthly aggregation (new cases *r *= 0.53; CI: 0.18–0.76; *p *< .001; hospitalizations *r *= 0.76; CI: 0.51–0.90; *p *< .001). The correlation with COVID-19 data did not improve with preselection, when only examinations in which clinicians suspected a pulmonary infection were included (new cases weekly *r *= 0.45; CI: 0.28–0.58; *p *< 0.001 and monthly *r *= 0.51; CI: 0.16–0.75; hospitalizations weekly *r *= 0.60; CI: 0.46–0.71; *p *< .001 and monthly *r *= 0.74; CI: 0.48–0.88; *p *< .001).

## Discussion

The goal of this study was to digitally and automatically label chest radiographs and chest CT reports as positive or negative for findings consistent with a pulmonary infection by means of text mining. The extracted labels allowed for calculation of the positive rate and the number needed to image to identify pulmonary infections, if clinically suspected. Chest radiographs and chest CT reports positive for pulmonary infections showed a clear and distinct seasonality pattern. This pattern matched moderately with regional influenza surveillance data from 2012 to 2020 (weekly data *r *= 0.45; monthly data *r *= 0.58) and the COVID-19 pandemic course from 2020 to 2022 (weekly new cases data *r *= 0.53; weekly hospitalizations data *r *= 0.65). Digitally labeling radiological examinations regarding their diagnosis would allow hospitals or individual departments to better understand their performance in diagnosis making and treatment decisions.

The overutilization of imaging, especially that of CT, is increasingly discussed given its impact on health care cost, workload, radiation exposure, and the environment [5, 22–24]. Raja et al created physician performance feedback reports for pulmonary embolism CT orders [15]. Reports included the rate of CT examinations with positive findings, derived from NLP-labeled radiology reports. This improved adherence to evidence-based guidelines for CT orders, but did not affect use and yield. In another study, a similar setup however decreased the use and increased the yield of CTs ordered to rule out pulmonary embolism in hospitalized patients [13]. Other studies investigating physicians’ clinical reasoning quality for radiology examination requests have relatively small sample size compared with overall imaging volumes and are therefore limited in their generalizability [7, 12]. Our approach allows for continuous and automated yield assessment of all imaging studies within a category, e.g., all chest CTs requested by the emergency department. The labeled data can be analyzed by means of business intelligence software in conjunction with other workflow data, irrespective of the practice setting.

The positive rate can be employed to gauge and calibrate the clinical reasoning of physicians ordering radiology examinations for a suspected disease, and to establish a sustained feedback loop [13–15]. Our results showed a lower number needed to image and a higher positive rate if clinicians suspected pneumonia in the examination request form, compared to data without preselection. Interestingly, preselection did not improve correlation of chest CT reports with COVID-19 data. This may be explained by a number of reasons, including the detection of non-COVID-19 pneumonia, prior antigen testing, or other factors which were not available for analysis in this study. While this demonstrates that clinical suspicion raises the pretest probability, there was also a considerable number of incidental pneumonia cases. These findings match the results of a study investigating clinical judgment accuracy in diagnosing pneumonia [25]. Fifty-seven percent of subjects with clinically suspected pneumonia also had positive findings on radiographs. However, out of all 140 patients with radiographically evident pneumonia, only 29% had been diagnosed clinically.

The positive rate itself is at first a neutral value which needs interpretation in the context of patient demographics, local hospital characteristics, and population health profiles. Ideally, a consensus on a reasonable range of positive rates can be established. Common risk score algorithms to assess the likelihood of a given diagnosis could be verified or refined to local or regional differences [26].

The ability to track the occurrence of a disease is essential to assess its extent and course. For most nations, there are tracking systems in place to monitor infectious diseases and other entities like cancer types [27]. However, data gathering can be challenging in terms of effort, cost, data quality, and accuracy [28]. The release of the Google flu tracker represented a novel approach, enabling to partially predict the forthcoming flu season by tracking search queries for flu like symptoms [9]. This demonstrates the existence of data signals potentially allowing for disease monitoring in an aggregated format. In a study similar to our work, Cury et al developed an NLP model which tracked positive CT imaging features of viral pneumonia and strongly correlated with the progression of the COVID-19 pandemic in the USA [16]. Their results demonstrate that large-scale data mining can turn previously neglected radiology report data into epidemiological information and support the need to render reports of any medical specialty machine readable.

Automated text mining of radiology reports allows for digital labeling and unlocks data mining capabilities to identify patterns such as seasonality, or correlation with clinical factors. By labeling chest radiographs and chest CTs, which represent standard clinical tests for pulmonary infections, it is possible to continuously track and monitor disease occurrence. Diseases appear to follow the dominant infection pathogen, be it the influenza or SARS-CoV-2 virus, as demonstrated. An interesting observation is the divergence of decreasing radiologically detected pulmonary infections and increasing new COVID-19 cases in our data (Fig. 4). This may represent a change in disease severity and a shift from lower to upper respiratory tract infections following the introduction of COVID-19 vaccinations in early 2021 [29].

By turning radiology reports into epidemiological data, hospitals or radiology departments can be employed as a real-time sensor with geographical resolution of population health. Combining this data on a local, regional, or national level should be easily achievable and would supplement the arsenal of tools for disease monitoring. While other approaches may have a reporting time lag and require additional efforts, i.e., general physicians manually providing data for national health care databases, radiology report information is already digitally present in real-time databases. Furthermore, reports have been signed by a board-certified physician and represent high-quality data sufficient for other physicians to act upon. Running a text mining algorithm requires minor effort and is fully automated, once established. Also, radiology imaging tests are fast and confirmative of a suspected diagnosis, and often represent the first and earliest reliable confirmation of a disease within the diagnostic workup.

Another application for labeled radiology report data is using the information as signal in the patient treatment pathway. For instance, once the diagnosis of a pulmonary infection in a patient with relevant comorbidities is made, an algorithm could prepare a comprehensive order within the hospital information system to be approved by a physician, i.e., for medication, a hospital bed for the estimated length of stay, or other care-associated aspects. In general, once this labeled data is available and patterns of seasonality or other occurrence-influencing cofactors have been identified, it should be possible to make predictions of future disease occurrence to allocate hospital resources accordingly. Our approach is in parallel to promising artificial intelligence–based disease-detection algorithms [30]. Nevertheless, NLP models analyze data created and signed off by physicians, compared to unsupervised image labeling, and can be shared and aggregated for data mining more easily than vast amounts of image data.

One aspect that facilitated report labeling by means of a regular expression algorithm is the consistent use of structured reporting and standardized terminology in our department [31–33]. Our reports often include a negation statement regarding common diagnoses such as pulmonary infections. Identifying this statement for labeling purposes is fairly easy compared to unstructured free-text reports with varying or non-existent expressions for the absence of a pathology [34]. Also, the labeling algorithm using regular expressions is transparent and verifiable, whereas neural network–based approaches are sometimes criticized as “black box” with results lacking scrutiny. Digital labeling of radiology reports may also benefit from recent developments in large language models [35].

Our study has limitations. A radiology report may not be true in the sense of ground truth or may contain a wrong diagnosis, but represents a standard clinical test for pulmonary infections. Referring physicians often form diagnoses and initiate patient treatment based on radiology report conclusions. The presented approach detects any pulmonary infection and is not specific to influenza or COVID-19 infections, but represents the dominant pulmonary infection at that time as demonstrated. It should be noted that CT was the institutional standard imaging test for suspected COVID-19 pneumonia between 2020 and 2022, which explains the observed shift from radiographs towards chest CTs. Algorithm accuracy was validated using a large sample from the data set. Nevertheless, isolated mislabeling may have occurred in case of misspelling or the use of a very unusual descriptive expression.

In conclusion, digitally labeling radiology reports with their diagnosis provides a new metric to track the trend of radiologically detectable diseases in real time. It allows to assess physicians’ clinical reasoning in ordering radiology examinations, the seasonality, and epidemiologic course of pulmonary infections, and offers a signal for workflow optimization for in-hospital patient care.

## Abbreviations

CI: Confidence interval
COVID-19: Coronavirus disease of 2019
CR: Chest radiographs
CT: Computed tomography
ICD: International Classification of Diseases
ILI: Influenza-like illness
NLP: Natural language processing
NNI: Number needed to image
RIS: Radiology information system

## Declarations

### Guarantor

The scientific guarantor of this publication is Tobias Heye.

### Statistics and biometry

No complex statistical methods were necessary for this paper.

### Study subjects or cohorts overlap

Study subjects or cohorts have not been previously reported, however data from this study was presented as a Research Presentation at the ECR 2022 in Vienna, Austria.

### Methodology

• retrospective

• experimental

• performed at one institution
